# Sociodemographic and Behavioral Factors Associated with HIV Vulnerability according to Sexual Orientation

**DOI:** 10.1155/2020/5619315

**Published:** 2020-01-24

**Authors:** Maria Aparecida A. O. Serra, Antoninho B. Milhomem, Samae B. Oliveira, Francisca Aline A. S. Santos, Roberta Araújo e Silva, Ana Cristina P. J. Costa, Maria da Conceição S. O. Cunha, Antônio Uelton A. Silva, Roberto Wagner J. F. Freitas, Márcio Flávio M. Araújo

**Affiliations:** ^1^Nursing Department, Centre for Social Science, Health and Technology, Federal University of Maranhão (UFMA), Imperatriz, 65900-410, Brazil; ^2^Nursing Department, Health Institute, University for International Integration of the Afro Brazilian Lusophony (UNILAB), Redenção, 62790-979, Brazil; ^3^Family Health Department, Oswaldo Cruz Foundation, 61760-000, Eusébio, Brazil

## Abstract

**Objective:**

To analyze sociodemographic and behavioral factors associated with vulnerability to HIV according to sexual orientation.

**Method:**

This is a cross-sectional study conducted using data on 3,818 people in the city of Imperatriz, Brazil, during 2015 and 2016. The survey's questionnaires addressed sociodemographic and behavioral variables. For the data analysis, association (chi-square test) and strength of association (odds ratio) were observed. A significance level of *p* < 0.05 and adjustment for age and gender were taken into consideration.

**Results:**

A substantial portion of the sample stated they were heterosexual (88.8%). These individuals demonstrated a lower chance of HIV infection (*p* < 0.001), sexually transmitted infections (*p* < 0.001), alcohol use (*p* < 0.001) and condom use (*p* < 0.001), compared to men who have sex with men and/or bisexuals. In this group, after adjusting for confounding variables, the factors associated with HIV infection were being male (*p* < 0.001), unmarried (*p* < 0.001), having completed higher education (*p* < 0.001) and boasting multiple sexual partners (*p* < 0.001).

**Conclusion:**

Behavioral and sociodemographic factors of vulnerability to HIV are predominant among men who have sex with men and/or are bisexual.

## 1. Introduction

The epidemic caused by the human immunodeficiency virus (HIV) represents a global, dynamic and unstable phenomenon. The nature of its occurrence in the different regions of the world depends, among other determinants, on individual and collective human behavior [1].

It is estimated that there are currently around 36.7 million individuals worldwide infected with HIV. In Brazil, between 2007 and June 2016, there were 136,945 reports of infection by the virus [2]. However, in Latin America, the annual number of new HIV infections among adults has remained stable since 2010. The number of people recently infected with HIV is significant in Latin American countries like Bolivia, Venezuela, Guatemala and Brazil [3]. Incidentally, just seven countries accounted for 90% of new cases of HIV infection in 2016 in Latin America. Of these, around one half of new infections (49%) occurred in Brazil, followed by Mexico with 13% [3].

Over the last ten years, the prevalence of HIV has been identified in some regions of Brazil as either in decline or remaining steady, however, the North and Northeast regions still exhibit a linear growing trend. Among the most noteworthy is the state of Maranhão, located in the Brazilian Northeast, where there was an increase of 82.2% in the detection of the disease. Between 1980 and 2016, there were 16,255 new cases of AIDS reported in this state [4].

The detection of new cases of HIV/AIDS has focused on specific groups of people with potential exposure to the virus, particularly through unprotected intercourse, such as sex workers, men who have sex with men (MSM), bisexuals, people with multiple partners, and alcohol/drug users [2, 5, 6].

Studies show that men's sexual proclivities have been responsible for the increased vulnerability to HIV. The data support the assertion that bisexual men and MSM individuals are more likely to acquire HIV than heterosexual men [7–9].

Understanding the factors related to people's sexual orientations, which are responsible for the increased risk of HIV infection, is important for the advocacy of minority groups and combating the HIV/AIDS epidemic. In addition, it is important to understand these associations based on a Social Determinants of Health (SDH) model as a theoretical framework. In Brazil, one of the SDH models adopted in healthcare studies related to people with HIV/AIDS, is the one proposed by Dahlgren and Whitehead [10].

This study's objective is to analyze sociodemographic and behavioral factors associated with vulnerability to HIV according to sexual orientation.

## 2. Materials and Methods

This is an epidemiological, observational and retrospective study, using an inductive approach. This strategy, also referred to as inductive reasoning, starts with the observations and theories proposed based on observation. Inductive research involves the search for patterns based on observation and the development of explanations for those patterns [11].

Ethical approval was obtained from the appropriate Research Ethics Committee under opinion no. 1.502.368. The participants in this study comprised individuals over 12 years of age, male, and female, with an active sex life in the twelve months preceding the survey.

The research was conducted between January and May 2017, at a healthcare unit specializing in STI/AIDS, in the city of Imperatriz, Maranhão, in northeastern Brazil. The city of Imperatriz has a population of approximately 252,320 and is the second largest in the state of Maranhão, in terms of population and also socioeconomic, political and cultural factors, having a medium human development index (HDI) of 0.731. Nevertheless, the municipality still faces problems in terms of the rates of illiteracy (9.7%) and basic sanitation (23%) [12, 13]. This information bears witness to the social setting of the city in which this study was conducted.

Data collection for this study was performed by nurses, previously trained for HIV/AIDS testing and counseling, in the period between 2015 and 2016. Consultations lasted approximately twenty minutes per participant and took place in a private setting. In the aforementioned period, the number of people contacting the health service (the location of the study) was 4,006. Thirty-eight people who did not satisfy the previously stipulated criteria were excluded from the survey.

Our data collection tool was developed based on new HIV case records, as recommended by the Brazilian Ministry of Health. Based on the data collection tool, we established—as a variable outcome—sexual orientation followed by HIV-positive status. The predictive variables investigated were then divided into sociodemographic factors (sex, age, marital status, skin color, education and remuneration) and clinical behavioral characteristics (presence of sexually transmitted infections, use of alcohol, tobacco and other drugs, number of sexual partners and the use of condoms).

According to the SDH model, the sociodemographic conditions in which people live have an impact on their health. The main social determinants of health are food, education, health services, environment, and the current socio-economic situation. In the analysis of the findings, we considered the two concentric layers of SDH proposed by Dahlgren and Whitehead, as follows: layer 1 (individual determinants: age and sex); and layer 2 (proximal determinants: individual behavior and lifestyle) [14].

The data were transferred to Microsoft Office Excel 2010, and the analyses were performed in the Statistical Package for the Social Sciences® (SPSS) program, version 22.0.

To begin with, the Kolmogorov-Smirnov test was applied for a weighting of the normality of the quantitative variables. Following this procedure, homoscedasticity was determined via Levene's test. Pearson's and/or Fisher's Chi-square tests, where applicable, were used to test the association between the variables, considering a significance level of *p* < 0.05. Furthermore, the odds ratio was calculated to estimate the effect of the variables evaluated.

Multiple logistic regression was performed to test independence between outcome and associated variables. The following outcome variables were chosen: HIV infection and independent variable are sociodemographic factors and risk behaviors for HIV infection, according to the stated sexual orientation. To determine independent risk factors for infection, logistic regression analysis was used.

The variables were selected from the simple multinomial logistic regression based on the Wald test (covariates *p*-value <0.20). The variables were introduced into the multiple model, one by one, in accordance with their statistical significance, observing the following blocks of variables: the first of the variables was sociodemographic, then followed by the behavioral ones. Variables remained in the multiple model when they were significant (*p* < 0.05) or when they were fitted to the model with regard to sex and age.

## 3. Results

### 3.1. MSM and Bisexual Individuals

As far as MSM and bisexuals are concerned, the predominant profile was male (92.0%), aged over 30 (73.5%), unmarried (85.7%), black/brown (76.8%), eighth-grade education or higher (85.2%), and the majority were engaged in some form of paid activity (58.2%).

In this study, we observed that MSM and bisexual individuals were more likely to be male (*p* < 0.0001, OR = 10.4, 95% CI = 7.34–14.9), unmarried (*p* < 0.0001, OR = 5.21, 95% CI = 3.94–6.89), and have attained a higher level of education (*p* < 0.0001, OR = 3.50, 95% CI = 2.65–4.61). On the other hand, this stratum was less likely to be in the over-30 age group (*p* < 0.0001, OR = 0.33, 95% CI = 0.26–0.42), as shown in Table 1. MSM and bisexual individuals were more likely to have multiple sexual partners (*p* < 0.0001, OR = 3.76, 95% CI = 2.93–4.81), as shown in Table 2.

In the group consisting of MSM and bisexuals infected with HIV, all were male (100%), aged under thirty (67.3%), unmarried (94.5%), black/brown skin color (87.3%), with eighth-grade education or higher (83.6%), and some form of paid employment (70.9%).

In the group consisting of MSM and bisexuals, HIV infection was most likely to happen to males (*p* = 0.01, OR = 1.16, 95% CI = 1.11–1.21), and also those with some form of paid employment (*p* = 0.041, OR = 1.88, 95% CI = 1.01–3.50). On the other hand, the lowest chances of HIV infection prevailed among married people (*p* = 0.044, OR = 0.31, 95% CI = 0.09–1.03) and among those who stated they were of white skin color (*p* = 0.048, OR = 0.44, 95% CI = 0.19–1.01), as shown in Table 3.

After multivariate analysis adjusted for sex and age, the behavioral variables were found not to be associated with HIV infection in the group of homosexuals/bisexuals.

MSM and bisexuals were more likely to have multiple sexual partners (*p* < 0.0001, OR = 1.608, 95% CI = 1.230–2.102). We did not find, in either group, a statistically significant association between being HIV-positive and a history of sexual abuse (heterosexual, MSM and bisexual individuals) (*p* = 0.911).

### 3.2. Heterosexual Individuals

With regard to the participants, we noted a predominance of males (56.8%) and those who declared themselves to be heterosexual. Participants' ages ranged from 12 to 84 years (SD ± 10.7).

Of the heterosexual individuals analyzed, the majority were male (52.4%), aged over thirty (51.7%), single (53.4%), black or brown (81.8%), with eighth-grade education or higher (62.2%), and some form of paid employment (63.8%).

We found that heterosexual individuals, in the last 12 months, were less likely to present with an STI (*p* < 0.0001, OR = 0.49, 95% CI = 0.38–0.62), to have used alcohol (*p* < 0.0001, OR = 0.62, 95% CI = 0.50–0.77), or to have used condoms (*p* < 0.0001, OR = 0.60, 95% CI = 0.46–0.77).

We also found that heterosexuals are less likely to be infected with HIV (*p* < 0.0001; OR = 0.25; 95% CI = 0.18–0.35).

Predominant among HIV-positive heterosexuals were male subjects (52.5%), aged over 30 (65.6%), married (50.8%), black/brown (82.8%), with eighth-grade education or higher (58.2%), and having some form of paid activity (56.6%). Among heterosexuals, the under-30 age group was the least likely to contract an HIV infection (*p* = 0.002, OR = 0.55, 95% CI = 0.37–0.80), while those (heterosexuals) with low levels of education were more likely to be infected with HIV (*p* < 0.0001, OR = 2.36, 95% CI = 1.64–3.41).

The result of the multivariate analysis, adjusted for age and sex, was that heterosexuals were more likely to use illicit drugs (*p* < 0.0001, OR = 2.021, 95% CI = 1.392–2.934), and less likely to have used condoms in the last 12 months (*p* = 0.001, OR = 0.623, 95% CI = 0.467–0.831), and were also less likely to be HIV-positive (*p* < 0.0001, OR = 0.232, 95% CI = 0.158–0.342).

Among heterosexuals, those who did not smoke cigarettes were more likely to be infected with HIV (*p* = 0.01, OR = 2.76, 95% CI = 1.17–6.53), as shown in Table 3. After multivariate analysis, adjusted for sex and age, heterosexuals who did not smoke cigarettes presented a higher risk of being HIV-positive (*p* = 0.02, OR = 2.63, 95% CI = 1.10–6.29).

When separately analyzing the two sexual orientation groups regarding the occurrence of HIV infection, from the clinical and/or behavioral variables, it was observed that heterosexuals who did not use the cigarette in the last twelve months had a higher chance being infected with the HIV virus (*p* = 0.01; OR = 2.76; 95% CI = 1.17–6.53), as shown in Table 3.

## 4. Discussion

### 4.1. MSM and Bisexual Individuals

According to layer 2 of the SDH model, which depicts proximal determinants, homosexuals and bisexuals were more vulnerable to being HIV-positive than heterosexuals (males). Brazilian epidemiological data have shown that, between 2007 and 2015, 59.4% of HIV infections occurred among homosexual or bisexual men. Moreover, this finding amounts to another global characteristic, namely that the number of infections among MSM individuals is on the rise [4, 15].

We believe that this finding resulted indirectly from an increase in the demand for counseling and testing services by the male population. The National Human Health Policy promoted by the Brazilian Ministry of Health in recent years may also have contributed toward these findings. Due to epidemiological changes, actions geared toward prevention, health education, and health promotion are important, especially when targeted at men who have sex exclusively with men and those who have sex with both men and women, as these groups—in addition to being in the minority—indulge in behavior that makes them prone to HIV infection [2, 6, 7].

In this study, multiplicity of partners was predominant among homosexuals and bisexuals. However, in this group, those who were married were less likely to be infected with HIV, while heterosexuals were less likely to have STIs or use alcohol. These findings support the current discussion in several studies on the vulnerability of homosexuals and bisexuals.

In general, unmarried individuals have exhibited a higher propensity to contract HIV infection [16–18]. However, previous studies conducted in Brazil, China and Canada have shown that unmarried MSM individuals and male bisexuals are more vulnerable to HIV infection [19–21].

Some authors have already pointed to the issue of multiplicity of partners among homosexuals and bisexuals, since it increases the chances of contracting STIs [21, 22]. In this study, the majority of MSM subjects and bisexuals had some form of paid employment (casual or formal work) and were in a lower age bracket than heterosexuals. This could partly explain the purchase of sex (sex workers) or drugs (licit and illicit) that favor the practice of unsafe sex. Both areas are problematical in Brazil, since they are unregulated and/or socially unacceptable activities.

Similarly, unprotected sex can be influenced by alcohol use, as it predisposes users to have multiple sexual partners and dispense with condoms [23, 24].

Among people living with HIV/AIDS, alcohol use can be a negative predictor for issues such as adherence to antiretroviral therapy, risky sexual activity and systemic physiological effects [25]. Thus, among those who are HIV positive, combating this habit is a priority for viral suppression [26].

Moreover, MSM and bisexual men have a greater number of sexual partners or even a greater propensity to indulge in sexual intercourse with strangers [27, 28]. Both of these situations may increase vulnerability to HIV infection. In addition, regarding the use of drugs, it is possible that this is the result of a regional cultural paradigm: people in the LGBT group are using both lawful and illicit drugs more frequently.

### 4.2. Heterosexual Individuals

Although heterosexuals in this study demonstrated reduced risk factors for HIV infection, they were also less likely to use condoms. In Latin America, the main way HIV is propagated is through unprotected sex, MSM, and transgender women being the most affected [26]. Condom use among Latinos depends on factors such as religion, relationship stability, level of education and income. For example, heterosexual Latino men prioritize the use of condoms in extramarital sex as opposed to when having sex with their wives [29]. Brazilians' concept of masculinity is that the man is the provider, so, to guarantee his health is to guarantee the family's subsistence [30].

Moreover, historically, the northeast region of Brazil (the focus of this study) has presented negative indicators related to women, such as *machismo*, domestic violence and femicide [31]. Accordingly, we believe that this finding reflected a gender issue. Furthermore, between heterosexual partners, there is a persistent feeling of trust, satisfaction, and intimacy, for which reason condoms may not be used in sexual relationships [32, 33].

These issues partly explain the fact that, in our study, heterosexuals used condoms infrequently but at the same time had less chance of contracting HIV.

Another peculiarity observed in the group of heterosexual participants was that those who did not use tobacco were more likely to be infected with HIV. These results suggested that infection in this group does not seem to be influenced by smoking, aside from reflecting the reduction in the number of smokers among the Brazilian population in recent years, due to the increase in the price of cigarettes, prohibition of tobacco advertising and the elimination of indoor, smoking-designated areas [34, 35].

Despite the greater use of illicit drugs and less frequent use of condoms, heterosexual individuals were less likely to be infected with HIV, according to this study. In fact, there are associations between the use of illicit drugs (injectables and non-injectables) and the risk of contamination with HIV [36, 37]. We believe that our findings derive from the fact that most subjects from the group of heterosexuals who participated in the study, are monogamous.

## 5. Limitations

This research had a number of limitations. The interviews conducted by the nurses addressed questions about behavior and/or clinical characteristics relating to sexuality. Therefore, because this is an intimate or embarrassing subject for some people, there is a probability of incomplete or false responses. Additionally, we did not analyze the association of the findings of HIV serology with information about participants' monthly income and/or socioeconomic classification or purchasing power.

Another point of view must be introduced as a potential limitation. Participants from this study were people who, of their own volition, approached an HIV-AIDS healthcare service; in other words, they are well aware of their risk for infection. People who do not seek or have not yet sought health services may present different or even more worrying behavior.

We emphasize that it is pertinent to conduct new studies, with different methodological approaches and/or scenarios (not related to health services) on this issue, as a way of helping health practitioners in primary healthcare, because, as we gain more knowledge on the peculiarities among heterosexuals, homosexuals and bisexuals, it will be possible to improve the epidemiological evaluation of HIV-AIDS and propose healthcare measures for the general population.

## 6. Conclusions

Heterosexual individuals were less likely to use condoms, to be infected with HIV, to have STIs and to use alcohol, than homosexuals and bisexuals.

MSM and bisexual individuals were more likely to be male, unmarried, have a higher level of education and have multiple sexual partners, when compared to heterosexuals.

## Figures and Tables

**Table 1:** Distribution of behavioral variables according to sexual orientation (*n* = 3,818).

Variables	Heterosexual (*N* = 3392)		Homosexual bisexual (*n* = 426)		*p* ^∗^ * value*	OR	95% CI
	*N*	%	*N*	%			
*STI in the last 12 months*							
Yes	433	12.8	98	23.0	**<0.0001**	0.49	0.38–0.62
No	2959	87.2	328	77.0			

*Alcohol in the last 12 months*							
Yes	1952	57.5	292	68.5	**<0.0001**	0.62	0.50–0.77
No	1440	42.5	134	31.5			

*Tobacco in the last 12 months*							
Yes	66	1.9	12	2.8	0.231	0.68	0.36–1.27
No	3326	98.1	414	97.2			

*Illegal drugs in the last 12 months*							
Yes	325	9.6	38	8.9	0.661	1.08	0.76–1.53
No	3067	90.4	388	91.1			

*Sexual partners in the last 12 months*							
One	1641	48.4	85	20.0	**<0.0001**	3.76	2.93–4.81
Multiple	1751	51.6	341	80.0			

*Used condoms for sex in the last 12 months*							
Yes	423	12.5	81	19.0	**<0.0001**	0.60	0.46–0.78
No	2969	87.5	345	81.0

*HIV serology*							
Reactive	122	3.6	55	12.9	**<0.0001**	0.25	0.18–0.35
Non-reactive	3270	96.4	371	87.1			

*N* = number; % = percentage; *p*^∗^ = Pearson's chi-square; OR = odds ratio; 95% CI = 95% confidence interval.

**Table 2:** List of sociodemographic variables according to the sexual orientation of individuals obtaining HIV-positive results (*n* = 177).

Variables	Heterosexual	*p* ^∗^ * value*	OR	95% CI	Homosexual/bisexual	*p* ^∗^ * value*	OR	95% CI
	*N* = 122				*N* = 55			
	*n* (%)				*n* (%)			
*Sex*								
Female	58 (47.5)	0.982	0.99	0.69–1.43	0 (0)	**0.01**	1.16	1.11–1.21
Male	64 (52.5)				55 (100)			

*Age*								
≤30	42 (34.4)	**0.002**	0.55	0.37–0.80	37 (67.3)	0.264	0.70	0.38–1.30
>30	80 (65.6)				18 (32.7)			

*Marital status*								
Married	62 (50.8)	0.339	1.19	0.83–1.71	3 (5.5)	**0.044**	0.31	0.09–1.03
Single	60 (49.2)				52 (94.5)			

*Skin color*								
White	21 (17.2)	0.769	0.93	0.57–1.50	7 (12.7)	**0.048**	0.44	0.19–1.01
Black/brown	101 (82.8)				48 (87.3)			

*Education level*								
<Eighth grade	71 (58.2)	**<0.0001**	2.36	1.64–3.41	9 (16.4)	0.724	1.14	0.53–2.48
≥Eighth grade	51 (41.8)				46 (83.6)			

*Paid employment*								
Yes	69 (56.6)	0.089	0.72	0.50–1.05	39 (70.9)	**0.041**	1.88	1.01–3.50
No	53 (43.4)				16 (29.1)			

*N* = number; % = percentage; *p*^∗^ = Pearson's chi-square; OR = odds ratio; 95% CI = 95% confidence interval.

**Table 3:** List of behavioral variables according to the sexual orientation of individuals obtaining HIV-positive results (*n* = 177).

Variables	Heterosexual	*p∗ value*	OR	95% CI	MSM/bisexual	*p∗ value*	OR	95% CI
	*N* = 122				*N* = 55			
	*n* (%)				*n* (%)			
*STI in the last 12 months*								
Yes	18 (14.8)	0.503	1.19	0.71–1.98	13 (23.6)	0.905	1.04	0.53–2.03
No	104 (85.2)				42 (76.4)			

*Alcohol in the last 12 months*								
Yes	73 (59.8)	0.602	1.10	0.76–1.59	39 (70.9)	0.686	1.13	0.61–2.11
No	49 (40.2)				16 (29.1)			

*Tobacco in the last 12 months*								
Yes	6 (4.9)	**0.01**	2.76	1.17–6.53	3 (5.5)	0.205	2.32	0.60–8.85
No	116 (95.1)				52 (94.5)			

*Illegal drugs in the last 12 months*								
Yes	16 (13.1)	0.177	1.44	0.84–2.47	6 (10.9)	0.579	1.29	0.51–3.26
No	106 (86.9)				49 (89.1)			

*Sexual partners in the last 12 months*								
One	64 (52.5)	0.358	1.18	0.82–1.70	6 (10.9)	0.072	0.45	0.18–1.09
Multiple	58 (47.5)				49 (89.1)			

*Condoms in the last 12 months*								
Yes	18 (14.8)	0.437	1.22	0.73–2.04	9 (16.4)	0.591	0.81	0.38–1.73
No	104 (85.2)				46 (83.6)			

## Data Availability

The data used to support the findings of this study are included within the article.
